# Prevalence and risk factors of vascular complications in type 2 diabetes mellitus: Results from discover Middle East and Africa cohort

**DOI:** 10.3389/fendo.2022.940309

**Published:** 2022-08-09

**Authors:** Khadija Hafidh, Rachid Malek, Khalid Al-Rubeaan, Adri Kok, Fahri Bayram, Akram Echtay, Viraj Rajadhyaksha, Ahmed Hadaoui

**Affiliations:** ^1^ Diabetes Unit, Rashid Hospital, Dubai, United Arab Emirates; ^2^ Internal Medicine, Setif University Hospital, Setif, Algeria; ^3^ Research and Scientific Centre, Sultan Bin Abdulaziz Humanitarian City, Riyadh, Saudi Arabia; ^4^ University of the Witwatersrand, Netcare Union and Clinton Hospitals, Alberton, South Africa; ^5^ Endocrinology and Metabolism, Erciyes University Faculty of Medicine, Kayseri, Turkey; ^6^ Endocrinology Division, Rafik Hariri University Hospital, Beirut, Lebanon; ^7^ Medical Affairs Department, AstraZeneca Middle East and Africa, Luton, United Kingdom; ^8^ Medical Affairs Department, AstraZeneca Algeria, Algiers, Algeria

**Keywords:** macrovascular complication, Middle East and Africa, microvascular complications, risk factors, type 2 diabetes, vascular complication

## Abstract

**Background:**

We evaluated the prevalence of vascular complications and associated risk factors in individuals with type 2 diabetes mellitus (T2DM) initiating second-line glucose-lowering therapy from the Middle East and Africa (MEA) cohort of the 3-year prospective DISCOVER study involving 15,992 patients in 38 countries.

**Methods:**

Baseline cross-sectional data collected from healthcare settings were used to assess micro and macrovascular complications prevalence as crude and age- and sex-standardised. The multi‐variable analysis assessed factors associated with these complications.

**Results:**

Of 3,525 enrolled patients (mean age: 54.3 ± 10.8 years), >40% had hypertension and hyperlipidaemia. Metformin monotherapy was the first-line therapy in 56.5%, followed by metformin+sulphonylurea (20.3%). Crude and standardised prevalence of microvascular complications were 17.7% and 16.9% (95% confidence interval [CI], 16.77‐16.98) and macrovascular complications were 10.7% and 8.7% (95% CI, 8.59–8.76). Factors significantly (p<0.05) associated with micro and macrovascular complications (odds ratios [95% CI]) were age (1.24 [1.12–1.39] and 1.58 [1.35–1.84]), male sex (1.33 [1.04‐1.70] and 1.71 [1.22–2.40]), hyperlipidaemia (1.33 [1.07-1.65] and 1.96 [1.46-2.63]) and hypertension (1.75 [1.40–2.19] and 2.84 [2.07-3.92]).

**Conclusion:**

A substantial burden of vascular complications with prominent risk factors in the MEA cohort calls for early preventive interventions.

## Introduction

Diabetes has seen exponential growth over the last two decades transcending all the socioeconomic and national boundaries to become a truly global epidemic (1). There has been a steady increase in the prevalence of diabetes with International Diabetes Federation (IDF) Middle East and North Africa (MENA) region having the highest age-adjusted comparative prevalence of diabetes (12.2%) in people aged 20 to 79 years with country-specific prevalence varying from 5.4% in Yemen to 22.1% in Sudan (2). MENA region has also reported the second-highest rate of rise, the highest‐adjusted mortality from non‐communicable disease and the highest diabetes-related disability‐adjusted life years (3).

In Africa, despite the lowest Age-adjusted diabetes prevalence and the highest proportion of undiagnosed diabetes among all IDF regions, South Africa registered a prevalence of 12.7% (2).

The most common form, type 2 diabetes mellitus (T2DM) is associated with various micro and macrovascular complications that negatively impact the quality of life and increase morbidity and mortality and account for the majority of the social and economic disease burden (4–8). T2DM remains underdiagnosed and is often detected incidentally. Therefore, the discovery of vascular complications is common at the time of diagnosis (9). Thus, the American Diabetes Association as well as the European Society of Cardiology and the European Association for the Study of Diabetes now advocate screening for co‐morbidities at diagnosis of T2DM and assessment of cardiovascular risk in individuals with diabetes and pre‐diabetes (10, 11).

A comprehensive understanding of the impact of patients’ characteristics, risk factors and management patterns in terms of clinical outcomes and T2DM progression is needed to improve the quality of interventions and reduce the burden of T2DM. In Middle East and Africa (MEA) countries as in many regions, such data remain scarce (12, 13). Thus, the DISCOVER (DISCOVERing Treatment Reality of Type 2 Diabetes in Real World Settings) study was conducted with an aim to address these knowledge gaps by providing real-world observational data on patient characteristics, disease management patterns and clinical outcomes over 3 years in individuals with T2DM who are initiating a second-line glucose-lowering therapy (GLT).

Considering the paucity of data in MEA countries on the prevalence of T2DM-related vascular complications at an early stage of the disease, we report the data from the MEA cohort of the DISCOVER study on the prevalence of microvascular and macrovascular complications and associated factors in individuals with T2DM, using a standardised methodology. Data from the DISCOVER overall population has been published earlier (14).

## Methods

### Study design

DISCOVER is a 3-year observational, prospective, longitudinal study carried out across 38 countries (ClinicalTrials.gov identifiers: DISCOVER in 37 countries [NCT02322762] and J‐DISCOVER in Japan [NCT02226822]). The methods and statistical analysis have been described in detail elsewhere (14–16). The study was approved by the relevant ethical committee of each country and the Institutional Review Board of each site and was conducted in accordance with the Declaration of Helsinki, the International Conference on Harmonisation of Good Clinical Practice and other applicable clinical guidelines.

We present the baseline cross-sectional analysis of 12 countries (Algeria, Bahrain, Egypt, Jordan, Kuwait, Lebanon, Oman, Saudi Arabia, South Africa, Tunisia, Turkey and United Arab Emirates [UAE]) representing the DISCOVER-MEA cohort.

Patients with T2DM, recruited from primary and specialist healthcare settings, initiating a second-line GLT after a first-line oral monotherapy or combination (dual or triple) therapy were enrolled in routine clinical practice. Patients were excluded if they had type 1 diabetes, were pregnant or using an injectable agent as first-line treatment. Informed consent was obtained from all the patients participating in the study at each site. The eligibility criteria are detailed in **Supplementary Table S1**. Patients meeting the inclusion/exclusion criteria were consecutively recruited in the study (site selection in terms of types and investigators’ specialties was made to achieve representativeness of the way T2DM is managed in each participating country). The patients underwent clinical assessments and receive standard medical care as determined by their treating physicians. No investigational treatment or experimental intervention was provided as a part of the study.

The reporting of results in the manuscript is as per the Strengthening the Reporting of Observational Studies in Epidemiology (STROBE) checklist (17).

### Data collection

Baseline data were collected using a standardized electronic case report form at the initiation of second‐line GLT and later transferred to a central database through a web-based data capture system. In addition to investigators’ characteristics, patients’ data regarding demographics, socioeconomic characteristics, physiological variables (blood pressure, pulse rate, body mass index [BMI]), laboratory parameters (glycated haemoglobin [HbA_1c_], total cholesterol, high‐density lipoproteins, low-density lipoproteins), duration of T2DM, first-line GLTs (received by patients before study baseline), second-line GLTs (prescribed at study baseline) and reason(s) for change in anti-diabetic therapy were collected at initiation of second-line therapy, co-medications, medical history and presence of comorbidities (including existing diabetes-related vascular complications) (14, 15).

Given the observational nature of the study, information with regard to clinical variables was collected as measured in routine clinical practice at each site, according to the local standard of care. Diagnosis and classification of complications relied on the judgement of investigators and there was no external independent adjudication of events.

The data on vascular complications included:

- Microvascular complications – nephropathy (presence of chronic kidney disease and/or albuminuria), retinopathy (history of retinopathy or retinal laser photocoagulation) and neuropathy (autonomic neuropathy, peripheral neuropathy and erectile dysfunction)
- Macrovascular complications – coronary artery disease (CAD) (history of CAD, angina, myocardial infarction [MI], percutaneous coronary intervention and coronary artery bypass grafting), cerebrovascular disease (stroke, transient ischaemic attack, carotid artery stenting and carotid endarterectomy), peripheral artery disease (history of peripheral artery disease including revascularisation procedures, diabetic foot and amputation), heart failure and implantable cardioverter-defibrillator use.

Patients who had experienced either micro or macrovascular disease prior to their T2DM diagnosis were not excluded. The overall and country-specific prevalence of micro and macrovascular complications and associated risk factors were evaluated.

### Statistical methods

The statistical analysis conducted was similar to that of the DISCOVER global cohort‐Vascular complications manuscript (14). Multi‐variate Imputation by Chained Equations (MICE) R package was used to perform the missing value imputation. All statistical analyses were carried out using the RStudio (Boston, MA, USA). The descriptive data (sex, tobacco smoking, co‐morbidities, co‐medications and first-line GLTs) were presented as numbers and percentages for categorical variables; mean (standard deviation [SD]) values were reported for continuous variables. Crude prevalence rates of micro and macrovascular diseases and those standardised for age and sex using a regression model are reported.

A multi‐variate analysis using a modified Poisson model determined the odds ratios (ORs) for the factors that were associated with micro and macrovascular complications. Variables included in the analysis were: age (per 10-year increment), sex, smoking status, HbA_1c_, duration of T2DM (per 1-year increment), hyperlipidaemia, and hypertension; p<0.05 was considered to be statistically significant.

## Results

### Baseline characteristics

The sociodemographic characteristics and clinical data of 3,525 patients included in the MEA cohort are presented in **Table 1**. About half (52.5%) were males; the mean age of patients was 54.3 ± 10.8 years for the overall MEA cohort with comparatively higher age in patients recruited from Lebanon (59.2 ± 10.3 years); it was lower in Oman (41.8 ± 8.5 years). The mean duration of T2DM since diagnosis was 6.2 ± 5.4 years with the highest duration reported in Kuwait (10.9 ± 7.3 years) and the lowest in Egypt (4.1 ± 3.7 years). The baseline mean HbA_1c_ for the overall MEA cohort was 8.7% ± 1.7 (median 8.3% [interquartile range: 7.6% to 9.4%]). Baseline HbA_1c_ did not vary significantly across the countries (mean HbA_1c_ range 8.3% to 9.0%). Among co‐morbidities, hypertension was reported in 43.2% (n=1,523) and hyperlipidaemia in 40.1% (n=1,413) of the patients in the overall MEA cohort. Bahrain and South Africa had a comparatively higher percentage of patients with hypertension (68.6% and 67.2%) while Kuwait (82.4%), Oman (77.4%), Bahrain (70.0%) and UAE (63.2%) had a higher percentage of patients with hyperlipidaemia compared to the overall cohort. Statins (42.3% [n=1,492]), angiotensin-converting enzyme inhibitors (ACEi; 33.6% [n=1,185]) and aspirin (22.4% [n=790]) were the most frequent co‐medications in the overall MEA cohort.

**Table 1:** Patient Demographics, Baseline Characteristics and Treatment Patterns, Overall and Country-wise.

Parameters	Overall (N=3,525)	Algeria(N=291)	Bahrain(N= 70)	Egypt(N=583)	Jordan(N=271)	Kuwait(N=51)	Lebanon(N=348)	Oman(N=31)	Saudi Arabia(N=519)	South Africa(N=519)	Turkey(N=534)	Tunisia(N=213)	UAE(N=95)
Sex, male, n (%)	1,850 (52.5)	141 (48.5)	45 (64.3)	330 (56.6)	164 (60.5)	40 (78.4)	198 (56.9)	22 (71.0)	284 (54.7)	163 (31.4)	269 (50.4)	128 (60.1)	66 (69.5)
Age, years, mean (SD)	54.3 (10.8)	55.3 (10.8)	54.2 (11.7)	52.9 (9.8)	53.8(11.3)	57.1 (9.5)	59.2 (10.3)	41.8 (8.5)	52.4 (11.0)	54.6 (11.4)	55.0 (10.0)	55.2 (9.6)	47.1 (10.1)
T2DM duration, years, mean (SD)	6.2 (5.4)	5.9 (4.8)	7.4 (5.0)	4.1 (3.7)	6.0 (5.8)	10.9 (7.3)	6.5 (5.2)	6.1 (4.9)	6.4 (5.2)	7.5 (6.0)	7.0 (5.9)	5.9 (4.9)	4.6 (3.6)
HbA_1c_ (%), mean (SD)	8.7 (1.7)	8.3 (1.6)	9.0 (2.0)	8.6 (1.4)	8.4 (1.6)	8.5 (1.4)	8.5 (1.6)	8.8 (2.0)	8.8 (1.7)	9.0 (2.1)	8.8 (1.8)	8.7 (1.5)	8.4 (1.7)
HbA_1c_ (%), median (IQR)	8.3% (7.6, 9.4)	7.9 (7.3, 9.0)	8.4 (7.7, 10.4)	8.4 (7.9, 9.1)	8.1 (7.4, 9.2)	8.3 (7.7, 9.0)	8.1 (7.5, 9.2)	8.8 (7.0, 9.6)	8.6 (7.6, 9.8)	8.4 (7.5, 10.1)	8.5 (7.5, 9.7)	8.4 (7.6, 9.4)	8.1 (7.4, 8.8)
BMI, kg/m^2^, mean (SD)	31.1 (5.9)	29.1 (4.6)	33.2 (5.9)	31.8 (5.1)	30.8 (5.0)	31.3 (5.4)	29.9 (4.6)	34.3 (7.5)	31.9 (6.6)	31.5 (6.8)	31.7 (6.4)	29.6 (5.4)	29.6 (6.5)
**Tobacco smoking, n (%)**													
Non-smoker	2,506 (73.1)	226 (78.5)	35 (50.0)	458 (79.9)	193 (73.7)	37 (77.1)	210 (61.0)	27 (87.1)	413 (80.0)	406 (79.3)	295 (59.8)	124 (63.9)	82 (86.3)
Ex-smoker	392 (11.4)	43 (14.9)	23 (32.9)	24 (4.2)	17 (6.5)	2 (4.2)	43 (12.5)	–	41 (7.9)	49 (9.6)	111 (22.5)	35 (18.0)	4 (4.2)
Current smoker	528 (15.4)	19 (6.6)	12 (17.1)	91 (15.9)	52 (19.8)	9 (18.8)	91 (26.5)	4 (12.9)	62 (12.0)	57 (11.1)	87 (17.6)	35 (18.0)	9 (9.5)
SBP, mm/Hg, mean (SD)	133.4 (16.6)	129.1 (14.9)	145.5 (16.9)	133.0 (14.9)	132.6 (16.4)	135.7 (12.2)	133.1 (14.5)	137.4 (17.9)	133.7 (17.5)	137.2 (19.8)	131.6 (15.8)	128.9 (13.0)	134.8 (15.8)
DBP, mm/Hg, mean (SD)	80.1 (10.0)	75.8 (8.9)	78.8 (9.4)	82.8 (8.6)	80.0 (10.0)	72.8 (11.0)	77.4 (8.0)	85.6 (13.1)	79.2 (10.9)	82.6 (10.5)	81.0 (9.9)	77.9 (8.6)	80.8 (9.7)
TC, (mg/dL), mean (SD)	191.3 (47.1)	174.5 (38.2)	177.8 (40.0)	201.6 (45.1)	188.7 (48.6)	162.7 (31.5)	189.7 (49.8)	195.4 (46.7)	190.0 (52.7)	186.8 (43.9)	207.5 (45.9)	188.5 (41.9)	176.4 (46.2)
Hypertension, n (%)	1,523 (43.2)	112 (38.5)	48 (68.6)	210 (36.0)	111 (41.0)	24 (47.1)	159 (45.7)	12 (38.7)	196 (37.8)	349 (67.2)	191 (35.8)	70 (32.9)	41 (43.2)
Hyperlipidaemia, n (%)	1,413 (40.1)	58 (19.9)	49 (70.0)	151 (25.9)	114 (42.1)	42 (82.4)	172 (49.4)	24 (77.4)	226 (43.5)	265 (51.1)	179 (33.5)	73 (34.3)	60 (63.2)
**Concomitant medication, n (%)**													
ACEi	1,185 (33.6)	114 (39.2)	42 (60.0)	170 (29.2)	93 (34.3)	20 (39.2)	111 (31.9)	16 (51.6)	156 (30.1)	200 (38.5)	156 (29.2)	69 (32.4)	38 (40.0)
β-Blocker	428 (12.1)	25 (8.6)	18 (25.7)	72 (12.3%)	37 (13.7)	6 (11.8)	63 (18.1)	3 (9.7)	45 (8.7)	61 (11.8)	65 (12.2)	27 (12.7)	6 (6.3)
Diuretic	478 (13.6)	36 (12.4)	16 (22.9)	42 (7.2)	37 (13.7)	8 (15.7)	36 (10.3)	1 (3.2)	29 (5.6)	187 (36.0)	58 (10.9)	23 (10.8)	5 (5.3)
High-intensity statin	427 (12.1)	9 (3.1)	15 (21.4)	84 (14.4)	55 (20.3)	–	76 (21.8)	1 (3.2)	75 (14.5)	44 (8.5)	25 (4.7)	28 (13.1)	15 (15.8)
Low-to-moderate Intensity statin	1,065 (30.2)	76 (26.1)	36 (51.4)	103 (17.7)	68 (25.1)	40 (78.4)	82 (23.6)	21 (67.7)	198 (38.2)	231 (44.5)	108 (20.2)	46 (21.6)	56 (58.9)
ASA	790 (22.4)	89 (30.6)	18 (25.7)	100 (17.2)	63 (23.2)	21 (41.2)	57 (16.4)	2 (6.5)	178 (34.3)	124 (23.9)	92 (17.2)	34 (16.0)	12 (12.6)
**First-line treatment, n (%)**													
Metformin (Met) monotherapy	1,990 (56.5)	225 (77.3%)	30 (42.9)	234 (40.2)	124 (45.8)	11 (21.6)	196 (56.3)	9 (29.0)	246 (47.4)	452 (87.1)	257 (48.1)	165 (77.5)	41 (43.2)
Met+SU+Thiaz (triple therapy)	30 (0.9)	4 (1.4%)	1 (1.4)	3 (0.5)	1 (0.4)	1 (2.0)	1 (0.3)	–	1 (0.2)	3 (0.6)	15 (2.8)	–	–
Other triple therapy	37 (1.0)	–	–	1 (0.2)	–	1 (2.0)	8 (2.3)	–	1 (0.2)	–	21 (3.9)	1 (0.5)	4 (4.2)
4 or 4+ therapy	8 (0.2)	–	–	–	–	1 (2.0)	5 (1.4)	–	1 (0.2)	–	1 (0.2)	–	–
SU mono	327 (9.3)	11 (3.8)	3 (4.3)	193 (33.2)	13 (4.8)	1 (2.0)	22 (6.3)	–	41 (7.9)	16 (3.1)	11 (2.1)	14 (6.6)	2 (2.1)
DPP4i monotherapy	22 (0.6)	1 (0.3)	3 (4.3)	4 (0.7)	1 (0.4)	–	5 (1.4)	–	3 (0.6)	–	2 (0.4)	2 (0.9)	1 (1.1)
Other monotherapy	21 (0.6)	2 (0.7)	–	4 (0.7)	2 (0.7)	–	3 (0.9)	–	–	–	6 (1.1)	3 (1.4)	1 (1.1)
Met+SU (dual therapy)	714 (20.3)	29 (10.0)	26 (37.1)	103 (17.7)	96 (35.4)	17 (33.3)	50 (14.4)	18 (58.1)	184 (35.5)	44 (8.5)	120 (22.5)	22 (10.3)	5 (5.3)
Met+DPP4i (dual therapy)	162 (4.6)	–	4 (5.7)	23 (4.0)	16 (5.9)	8 (15.7)	31 (8.9)	1 (3.2)	23 (4.4)	1 (0.2)	32 (6.0)	2 (0.9)	21 (22.1)
Met+other (dual therapy)	79 (2.2)	18 (6.2)	–	3 (0.5)	6 (2.2)	3 (5.9)	3 (0.9)	–	–	–	44 (8.2)	1 (0.5)	1 (1.1)
Met+SU+DPP4i (triple therapy)	91 (2.6)	–	3 (4.3)	6 (1.0)	7 (2.6)	7 (13.7)	21 (6.0)	2 (6.5)	16 (3.1)	1 (0.2)	20 (3.7)	–	8 (8.4)
Other dual therapy	43 (1.2)	1 (0.3)	–	8 (1.4)	5 (1.8)	1 (2.0)	3 (0.9)	1 (3.2)	3 (0.6)	2 (0.4)	5 (0.9)	3 (1.4)	11 (11.6)

Missing data are not included.

ACEi, angiotensin-converting enzyme inhibitors; ASA, acetylsalicylic acid; BMI, body mass index; DBP, diastolic blood pressure; DPP4i, dipeptidyl peptidase-4 inhibitor; HbA_1c_, glycated haemoglobin; IQR, interquartile range; SBP, systolic blood pressure; SD, standard deviation; SU, sulphonylurea; T2DM, type 2 diabetes mellitus; TC, total cholesterol; Thiaz, thiazolidinediones; UAE, United Arab Emirates.

As first-line GLTs, more than half (56.5% [n=1,990]) of the patients received metformin monotherapy, followed by dual therapy of metformin+sulphonylurea (SU) in 20.3% (n=714). In a country-wise analysis of first-line GLT, except for Oman (29.0%) and Kuwait (21.6%), metformin monotherapy was commonly prescribed as first-line GLT (range: 40.2% to 87.1%). In Oman and Kuwait, dual therapy of metformin+SU was the most prescribed treatment (58.1% and 33.3%) as first-line GLT, respectively. Except for Egypt, metformin+SU was the second most common GLT (range: 10.0% to 58.1%). In Egypt, SU monotherapy was the second most commonly prescribed (33.2%) GLT.

### Prevalence of microvascular complications

The crude prevalence of microvascular complications in the overall MEA cohort was 17.7%; the age- and sex-standardised prevalence was 16.9% (95% confidence interval [CI], 16.77‐16.98) (**Table 2**). The country-wise crude prevalence of microvascular complications is shown in **Supplementary Table S2** and the age- and sex-standardized prevalence are shown in Table 3. The age- and sex-standardized prevalence of microvascular disease was highest in Kuwait 28.6% (95% CI, 23.65-34.15), closely followed by Oman 28.0% (95% CI, 21.16‐35.97) and was lowest in Jordan 7.9% (95% CI, 7.50-8.37). Peripheral neuropathy was the most prevalent microvascular complication in the overall MEA cohort (crude prevalence: 8.2%; age- and sex-standardised prevalence: 7.8% [95% CI, 7.72‐7.87]). It was the most prevalent complication in Egypt (13.3%), Turkey (10.9%), Saudi Arabia (10.4%), Algeria (8.6%), South Africa (2.9%) and Jordan (2.8%). In Kuwait and Bahrain, retinopathy (15.0% and 4.3%) was the most prevalent complication. albuminuria (28.0%, 9.9% and 5.6%) in Oman, Tunisia and Lebanon respectively, while chronic kidney disease (CKD; 3.2%) in UAE, were the most prevalent.

**Table 2:** Crude and Age- and Sex-Standardised Prevalence of Microvascular and Macrovascular Complications and Related Procedures at Baseline in Overall Cohort.

Event	Crude prevalence (n [%]) (N=3,525)	Age- and sex-standardised prevalence (% [95% CI]) (N=3,525)
Microvascular disease	623 (17.7)	16.9 (16.77-16.98)
CKD	61 (1.7)	1.0 (1.00-1.05)
Albuminuria	120 (3.4)	3.2 (3.16-3.26)
Retinopathy	123 (3.5)	2.7 (2.62-2.71)
Retinal laser photocoagulation	17 (0.5)	0.3 (0.32-0.36)
Peripheral neuropathy	288 (8.2)	7.8 (7.72-7.87)
Autonomic neuropathy	32 (0.9)	0.7 (0.68-0.73)
ED	161 (4.6)	–
Macrovascular disease	377 (10.7)	8.7 (8.59-8.76)
HF	41 (1.2)	0.8 (0.73-0.78)
CAD	299 (8.5)	6.8 (6.68 -6.83)
Angina	80 (2.3)	1.9 (1.82-1.90)
MI	87 (2.5)	1.8 (1.75- 1.83)
PCI	113 (3.2)	2.5 (2.50 – 2.60)
CABG	36 (1.0)	0.4 (0.38-0.42)
Stroke	39 (1.1)	0.8 (0.75-0.80)
TIA	22 (0.6)	0.5 (0.51-0.55)
Carotid stenting	4 (0.1)	0.0
Carotid endarterectomy	1 (0.0)	0.0
PAD	18 (0.5)	0.4 (0.33-0.37)
Diabetic foot	21 (0.6)	0.5 (0.48-0.52)
Amputation	12 (0.3)	0.3 (0.27-0.30)
Defibrillator use	0 (0.0)	0.0

Percentages calculated for all patients with data available; missing data are not included.

CAD, coronary artery disease; CABG, coronary artery bypass graft; CKD, chronic kidney disease; CI, confidence interval; ED, erectile dysfunction; HF, heart failure; MI, myocardial infarction; PAD, peripheral artery disease; PCI, percutaneous coronary intervention; TIA, transient ischaemic attack.

### Prevalence of macrovascular complications

In the overall MEA cohort, for macrovascular complications, the crude prevalence was 10.7% and the age- and sex- standardized was 8.7% (95% CI, 8.59‐8.76) (**Table 2**). The crude as well as age- and sex-standardized prevalence was the highest for CAD (8.5% and 6.8% [95% CI, 6.68 to 6.83]) in the overall MEA cohort. The crude country-wise prevalence of macrovascular complications is shown in **Supplementary Table S2** and the age- and sex-standardized prevalence are shown in Table 3. The age- and sex-standardized prevalence of macrovascular complications was the highest in Turkey at 14.4% (95% CI, 14.16‐14.71). CAD was the most prevalent complication in all countries (range: 2.7%‐11.6%).

### Multi‐variable analysis

**Figure 1:**
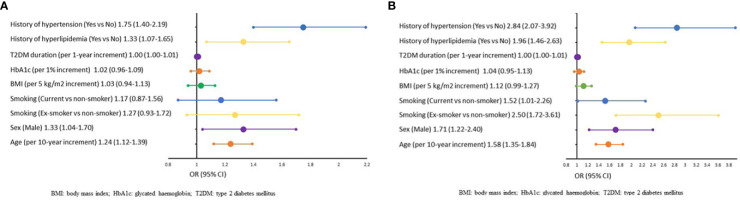
**(A)**, Multi‐variable Analysis of Factors Associated With Microvascular Complications. BM, body mass index; HbA_1c_, glycated haemoglobin; T2DM, type 2 diabetes mellitus. **(B)**, Multi‐variable Analysis of Factors Associated With Macrovascular Complications BMI, body mass index; HbA_1c_, glycated haemoglobin; T2DM, type 2 diabetes mellitus.

**Table 4** and **Figures 1A, B** show the factors associated with micro and macrovascular complications. In the multi‐variable analysis, similar factors were associated with a higher prevalence of both micro and macrovascular complications. The factors significantly associated (p<0.05) were age per 10-year increment (microvascular: OR 1.24 [95% CI, 1.12‐1.39], macrovascular: OR 1.58 [95% CI, 1.35‐1.84]); male sex (microvascular: OR 1.33 [95% CI, 1.04‐1.70], macrovascular: OR 1.71 [95% CI, 1.22‐2.40]), history of hyperlipidaemia (microvascular: OR 1.33 [95% CI, 1.07‐1.65], macrovascular: OR 1.96 [95% CI, 1.46‐2.63]) and history of hypertension (microvascular: OR 1.75 [95% CI, 1.40‐2.19], macrovascular: OR 2.84 [95% CI, 2.07-3.92]). Apart from these factors, current smokers (OR 1.52 [95% CI, 1.01‐2.26]) and ex-smokers (OR 2.50 [95% CI, 1.72 to 3.61]) showed significant association with macrovascular complications (both p<0.05) compared with non-smokers. A multi‐variable analysis was not performed for individual countries due to small sample size.

**Table 4:** Multi‐variable Analysis for Factors Associated With Microvascular and Macrovascular Complications.

Factors		MicrovascularOR (95% CI)	p-value	MacrovascularOR (95% CI)	p-value
Age (per 10-year increment)		1.24 (1.12-1.39)	<0.001	1.58 (1.35-1.84)	<0.001
Sex	Male	1.33 (1.04-1.70)	0.021	1.71 (1.22-2.40)	0.002
Smoking (vs non-smoker)	Ex-smoker	1.27 (0.93-1.72)	0.135	2.50 (1.72-3.61)	<0.001
	Current smoker	1.17 (0.87-1.56)	0.300	1.52 (1.01-2.26)	0.042
BMI (per 5 kg/m^2^ increment)		1.03 (0.94-1.13)	0.514	1.12 (0.99-1.27)	0.069
HbA_1c_ (per 1% increment)		1.02 (0.96-1.09)	0.465	1.04 (0.95-1.13)	0.372
T2DM duration (per 1-year increment)		1.00 (1.00-1.01)	<0.001	1.00 (1.00-1.01)	<0.001
History of hyperlipidaemia (Yes vs No)		1.33 (1.07-1.65)	0.010	1.96 (1.46-2.63)	<0.001
History of hypertension (Yes vs No)		1.75 (1.40-2.19)	<0.001	2.84 (2.07-3.92)	<0.001

p-value of <0.05 was considered to be significant.

BMI, body mass index; CI, confidence interval; HbA_1c_, glycated haemoglobin; OR, odds ratio; T2DM, type 2 diabetes mellitus.

## Discussion

This analysis focused on the prevalence of vascular complications and their potentially associated factors in individuals with T2DM initiating a second-line GLT in the MEA cohort of the DISCOVER study. We found the overall crude prevalence of microvascular and macrovascular complications to be 17.7% and 10.7%. The age- and sex-standardised prevalence of microvascular and macrovascular complications was 16.9% (95% CI, 16.77‐16.98) and 8.7% (95% CI, 8.59‐8.76), respectively. The prevalence varied markedly across the countries in MEA (**Table 3A** and **Table 3B**). Among microvascular complications, peripheral neuropathy was the most prevalent one with age- and sex-standardised prevalence of 7.8% (95% CI, 7.72‐7.87). CAD was the most prevalent macrovascular complication with age- and sex-standardised prevalence of 6.8% (95% CI, 6.68 to 6.83).

**Table 3A:** Age- and Sex-standardised Prevalence of Microvascular and Macrovascular Complications, Country-wise.

Event	Algeria(N=291)	Bahrain(N=70)	Egypt(N=583)	Jordan(N=271)	Kuwait(N=51)	Lebanon(N=348)
**Overall Microvascular disease, (95% CI)**	20.8 (18.53-23.25)	16.6 (14.20-19.22)	20.4 (20.10 -20.68)	7.9 (7.50-8.37)	28.6 (23.65-34.15)	12.7 (12.24-13.10)
**CKD**	2.9 (2.07-3.95)	2.1 (1.38-3.05)	0.0	0.2 (0.17-0.32)	0.0	0.0
**Albuminuria**	4.6 (3.61-5.92)	0.0	1.4 (1.27-1.44)	1.6 (1.38-1.83)	0.1 (0.00-1.00)	5.6 (5.33-5.89)
**Retinopathy**	2.0 (1.25-3.06)	4.3 (3.23-5.63)	1.9 (1.82-2.00)	1.6 (1.38-1.76)	15.0 (11.60-19.29)	0.8 (0.68-0.89)
**Retinal laser photocoagulation**	0.6 (0.31-1.18)	0.0	0.2 (0.16-0.21)	0.0	0.0	0.0
**Peripheral Neuropathy**	8.6 (7.20-10.31)	0.3 (0.11-0.71)	13.3 (13.07-13.56)	2.8 (2.54-3.03)	0.2 (0.00-1.00)	1.2 (1.12-1.37)
Autonomic Neuropathy	0.0	0.0	2.0 (1.90-2.10)	0.3 (0.21-0.36)	0.0	0.0
**Overall macrovascular disease, n (%)**	5.0 (3.88-6.37)	8.7 (7.14-10.65)	8.0 (7.77-8.16)	8.9 (8.42-9.40)	3.8 (1.13-11.81)	7.9 (7.52-8.27)
HF	0.0	0.0	0.5 (0.50-0.59)	0.2 (0.12-0.23)	0.0	0.9 (0.75-0.99)
CAD	3.2 (2.31-4.52)	4.5 (3.46-5.90)	6.0 (5.88-6.22)	7.4 (6.95-7.81)	3.8 (1.13-11.81)	7.0 (6.65-7.37)
Angina	0.4 (0.14-0.85)	0.0	2.1 (2.04-2.24)	0.0	0.0	0.0
MI	0.2 (0.02-1.31)	0.0	0.8 (0.75-0.87)	1.6 (1.45-1.83)	0.1 (0.00-1.00)	1.4 (1.24-1.52)
PCI	0.0	0.0	2.1 (1.96-2.18)	2.9 (2.68-3.19)	5.5 (3.72- 8.10)	2.6 (2.36-2.78)
CABG	0.0	0.0	0.0	0.0	0.0	0.6 (0.48-0.66)
Stroke	0.4 (0.16-0.92)	0.0	0.3 (0.30-0.38)	0.4 (0.31-0.52)	0.0	0.0
TIA	0.4 (0.16-0.95)	0.2 (0.08-0.62)	0.3 (0.25-0.32)	0.0	0.0	0.0
Carotid stenting	**0.0**	**0.0**	0.0	0.0	0.0	0.0
Carotid Endarterectomy	**0.0**	**0.0**	0.0	0.0	0.0	0.0
PAD	0.0	0.0	0.9 (0.82-0.94)	0.5 (0.44-0.67)	0.0	0.0
Diabetic foot	0.4 (0.16-0.92)	1.3 (0.81-2.18)	0.3 (0.29-0.37)	0.0	0.0	0.0
Amputation	0.0	0.0	0.0	0.0	0.0	0.0
Defibrillator use	0.0	0.0	0.04	0.0	0.0	0.0

CAD, coronary artery disease; CABG, coronary artery bypass graft; CKD, chronic kidney disease; CI, confidence interval; ED, erectile dysfunction; HF, heart failure; MI, myocardial infarction; PAD, peripheral artery disease; PCI, percutaneous coronary intervention; TIA, transient ischaemic attack.

**Table 3B:** Age- and Sex-Standardised Prevalence of Microvascular and Macrovascular Complications, Country-wise.

Event	Oman(N=31)	Saudi Arabia(N=519)	South Africa(N=519)	Turkey(N=534)	Tunisia(N=213)	United Arab Emirates(N=95)
**Overall Microvascular disease, (95% CI)**	28.0 (21.16-35.97)	19.5 (18.68-20.30)	8.6 (8.39-8.82)	19.4 (19.09 -19.70)	18.2 (17.47-18.99)	9.1 (7.84-10.47)
**CKD**	0.0	0.0	1.3 (1.23-1.41)	0.8(0.74-0.86)	1.3 (1.13-1.57)	3.2 (2.50-4.08)
**Albuminuria**	28.0 (21.16-35.97)	2.2 (1.86-2.64)	0.8 (0.70-0.83)	1.3 (1.17-1.35)	9.9 (9.30-10.50)	0.0
**Retinopathy**	0.0	2.6 (2.25-2.95)	1.6 (1.54-1.75)	4.6 (4.49-4.82)	1.6 (1.33-1.91)	1.9 (1.39-2.62)
**Retinal laser photocoagulation**	0.0	0.0	0.0	0.6 (0.56-0.69)	1.0 (0.77-1.16)	0.0
**Peripheral neuropathy**	0.0	10.4 (9.83 -11.05)	2.9 (2.78-3.12)	10.9 (10.66-11.14)	3.8 (3.42-4.21)	2.4 (1.77-3.24)
Autonomic neuropathy	0.0	0.6 (0.46-0.81)	0.0	0.2 (0.17-0.23)	0.0	0.0
**Overall macrovascular disease, (95% CI)**	0.0	7.5 (6.89-8.15)	7.5 (7.25-7.68)	14.4 (14.16-14.71)	6.1 (5.66-6.62)	4.2 (3.33-5.21)
HF	0.0	0.9 (0.73 -1.21)	0.2 (0.17-0.26)	1.1 (1.00-1.15)	0.0	0.0
CAD	0.0	5.2 (4.72-5.82)	6.3 (6.08-6.47)	11.6 (11.36-11.85)	4.2 (3.78-4.57)	2.7 (2.03-3.46)
Angina	0.0	2.0 (1.73-2.33)	2.4 (2.23-2.50)	2.9 (2.75-3.00)	1.1 (0.88-1.30)	0.0
MI	0.0	0.6 (0.40-0.92)	3.6 (3.49-3.79)	2.5 (2.41-2.65)	0.0	0.0
PCI	0.0	2.3 (1.98-2.75)	0.2 (0.21-0.28)	5.6 (5.46-5.81)	1.3 (1.10-1.59)	0.0
CABG	0.0	0.0	0.4 (0.38-0.47)	1.1 (1.01-1.21)	0.0	0.0
Stroke	0.0	1.2 (0.97-1.58)	0.0	0.6 (0.58-0.69)	0.1 (0.03-0.15)	0.0
TIA	0.0	0.0	0.0	0.3 (0.27-0.35)	0.8 (0.67-1.01)	0.0
Carotid stenting	0.0	0.3 (0.22-0.44)	0.0	0.0	0.0	0.0
Carotid endarterectomy	0.0	0.0	0.0	0.0	–	0.0
PAD	0.0	0.0	0.3 (0.25-0.33)	0.0	–	0.0
Diabetic foot	0.0	0.0	0.0	1.0 (0.87 -1.04)	–	0.0
Amputation	0.0	0.0	0.7 (0.61-0.73)	0.0	–	0.0
Defibrillator use	0.0	0.0	0.0	0.0	–	0.0

CAD, coronary artery disease; CABG, coronary artery bypass graft; CKD, chronic kidney disease; CI, confidence interval; ED, erectile dysfunction; HF, heart failure; MI, myocardial infarction; PAD, peripheral artery disease; PCI, percutaneous coronary intervention; TIA, transient ischaemic attack.

The standardised prevalence of vascular complications in MEA was similar to DISCOVER global data, which reported a prevalence of microvascular complications at 17.9% (95% CI, 17.3‐18.6) and macrovascular complications at 9.2% (95% CI, 8.7‐9.7) that varied across the regions (14).

This manuscript explored the crude prevalence of cardiovascular disease (CVD) in the MEA region, which is less than what has been observed in western countries as shown by global data, warranting further explanation (14). Of the 38 countries participating in the DISCOVER study, the MEA cohort comprised 12 countries accounting for almost one-fifth (22.1%) of the total DISCOVER population. Overall, the baseline characteristics and patients’ sociodemographics were similar across all the countries. Oman had a younger population (41.8 ± 8.5 years) compared with the MEA cohort (54.3 ± 10.8 years) as well as the global DISCOVER cohort (57.2 ± 12.0 years). The duration of T2DM was of 6.2 years, which was comparatively higher than the DISCOVER global data (5.6 years) (14). The duration of T2DM was the highest in Kuwait (10.9 ± 7.3 years) and lowest in Egypt (4.1 ± 3.7 years).

In line with the global data and as expected in patients who were moving to second-line therapy, mean HbA_1c_ was above the target of 7.0% recommended by international (11, 18–20) as well as local guidelines (21–23). Based on the median HbA_1c_ (8.3% [interquartile range: 7.6% to 9.4%]) values, more than 50% of patients for whom HbA_1c_ was reported had levels above 8.0%, and >25% had a measurement higher than 9.0%. This suggests suboptimal glycaemic control and delayed treatment intensification increasing the risk of vascular complications.

More than 40% of patients in the MEA cohort had hyperlipidaemia and hypertension and most of them were prescribed statins and ACEis. A similar prevalence of hypertension and hyperlipidaemia was observed in cross-sectional studies from Saudi Arabia, Turkey and Jordan (24–27). In the VISION study, the prevalence of hypertension and dyslipidaemia in patients initiating insulin in the MEA region were 42.9% and 43.5% (28). In South Africa, analysis using medicine claims data reported a prevalence of 35.0% and 45.6% for hyperlipidaemia and hypertension respectively (29). All these studies confirm the high prevalence of two major risk factors for CVD in individuals with T2DM – hypertension and dyslipidaemia.

The prevalence of micro and macrovascular complications in people with T2DM has been evaluated in several studies. In the international, open-label, observational A1chieve study in more than 66,000 individuals with T2DM initiated on insulin across 28 countries from 4 continents (Asia, Africa, Europe and South America), the overall prevalence of micro and macrovascular complications was 53.5% and 27.2%, respectively compared with 65.8% and 28.7% for micro and macrovascular complications in the Middle East subgroup (30). Also, the Middle East cohort had younger patients with micro and macrovascular complications compared with the global A1chieve cohort. In a review of prevalence studies and cross‐sectional surveys published between 2007 to 2017, estimating the prevalence of CVDs in individuals with T2DM, though the prevalence of CVD in the MENA region was lower than global data (26.9% vs 32.2%), the prevalence of CAD (27.4% vs 21.2%) and MI (11.4% vs 10.0%) was higher (31). In the CAPTURE study evaluating the prevalence of CVD in individuals with T2DM, the prevalence of CVD were 18.0% in Saudi Arabia and 31.2% in Turkey (32).

In a retrospective cohort study in the United States reporting the prevalence of micro and macrovascular complications in 135 199 patients with newly diagnosed T2DM, the prevalence of CKD was 12.3%, peripheral neuropathy was 3.8% and composite CVD was 3.3% at diagnosis (33). A study from Italy in patients with newly diagnosed T2DM reported CVD in 11.2%, neuropathy in 18.6%, CKD in 8.8% and albuminuria in 13.2% of cases (34). In our MEA cohort, though the prevalence of CKD was not more than 1.0%, albuminuria was reported in 3.2% of patients. The prevalence of albuminuria was the highest in Oman (27.9%); however, due to the small sample size from Oman (n=31), the generalisation of this data is limited. Studies from the Middle East, Gulf and Africa region are limited; however, data from South Africa published in 1997 reported a prevalence of 15.6% for proliferative and preproliferative retinopathy, 27.6% for peripheral neuropathy and 36.7% for microalbuminuria after a mean diabetes duration of 8 years (35). Results from Saudi National Diabetes Registry reported a prevalence of 10.8% for nephropathy and 19.7% for retinopathy (36, 37). In the Gulf DiabCare survey conducted in Saudi Arabia, Kuwait and the UAE, from 1,290 individuals with T2DM, 34.9% of patients had neuropathy, 29.9% of patients had retinopathy, and <10% of patients had CVD and end-stage renal failure (38). In a study from Qatar, the prevalence of microvascular complications was 48.4% (500 of 1,034 patients) with 13.5% of patients presenting with more than one complication (39). Individuals with complications had lower age of onset, higher duration of T2DM and higher HbA_1c_ compared with individuals without complications (all p<0.0001).

Results of our correlation analysis revealed a relationship between vascular complications and patients’ characteristics – age, male sex, smoking status, BMI, HbA_1c_, history of hyperlipidaemia and hypertension but the association was only significant with higher age, male sex, current/ex-smoking status, and history of hyperlipidaemia and hypertension (p<0.05). Micro and macrovascular complications were positively and significantly associated with the same risk factors but the ORs were higher for macrovascular complications. Additionally, we found that BMI and HbA_1c_ were not significantly associated with either micro and macrovascular complications similar to that reported for the global DISCOVER study population macrovascular analysis but contrasts with microvascular one where HbA_1c_ was found to be significantly associated (14). In a study from Qatar, similar risk factors for microvascular complications were reported along with other factors like family history, time in formal education, oral GLTs and hypertension (39).

In the A1chieve study, a multi‐variate analysis of the factors positively associated with micro and macrovascular complications were age, BMI, diabetes duration, total cholesterol, triglycerides and systolic blood pressure. High-density lipoproteins were negatively associated with macrovascular complications (30).

DISCOVER-MEA is the first prospective real-world study in this region evaluating the prevalence and risk factors for vascular disorders in patients with T2DM who have progressed to second-line therapy. Although the prevalence of micro and macrovascular disorders was similar to DISCOVER global data, the increasing prevalence of T2DM in the MEA region should be accounted for, for future strategies for the holistic management of T2DM. Outcomes-driven management of T2DM is needed for decreasing the risk of vascular complications.

The limitations of our study are those of real-world observational studies. The diagnosis of vascular disorders was not standardised across countries and thus, bias might have been introduced due to judgment by treating physicians. Also, small sample sizes for some countries did not allow for the exact interpretation of age- and sex-standardised prevalence and a multi‐variable analysis. Healthcare utilization is dependent on socioeconomic status which may impact access and hence reporting of vascular complications; however, this analysis was not undertaken for this manuscript because of limited data from most of the participating countries.

## Conclusion

Despite having a short duration of T2DM, the MEA cohort had a substantial burden of vascular complications in patients mainly treated but suboptimally controlled with metformin and metformin+SUs. This increasing burden should be considered for formulating the strategies to manage T2DM. Early assessment of risk factors, cardiovascular risk status and preventive strategies should be adopted for effective management of T2DM in the MEA region.

## Data availability statement

The raw data supporting the conclusions of this article will be made available by the authors, without undue reservation.

## Ethics statement

The study was reviewed and approved by the relevant ethical committee of each country and the Institutional Review Board of each site and was conducted in accordance with the Declaration of Helsinki, the International Conference on Harmonisation of Good Clinical Practice, and other applicable clinical guidelines. The patients/participants provided their written informed consent to participate in this study.

## Author contributions

The general content of the manuscript was agreed upon by all authors. All authors approved the final version of the manuscript before its submission. An AstraZeneca team reviewed the manuscript during its development and was allowed to make suggestions. The final content was determined by all the authors. The precise role of each author is as follows • Conceptualization: KH, RM, KR, AK, FB, AE, VR, AH • Data curation: KH, RM, KR, AK, FB, AE, VR, AH • Investigation: KH, RM, KR, AK, FB, AE • Methodology: KH, RM, KR, AK, FB, AE, VR, AH • Validation: KH, RM, KR, AK, FB, AE, VR, AH • Visualization, Writing, Review & editing: KH, RM, KR, AK, FB, AE, VR, AH.

## Funding

The DISCOVER study program is funded by AstraZeneca. DISCOVER is a non‐interventional study and no drugs were supplied or funded.

## Acknowledgments

The authors would like to thank all investigators and patients who participated in the DISCOVER study program. Medical writing support was provided by Prajakta Nachane, LabCorp Scientific Services & Solutions Pvt. Ltd, and was funded by AstraZeneca.

## Conflict of interest

KH has received speaker fees and research support from AstraZeneca, Boehringer Ingelheim, Servier, Sanofi, Merck & Co., Novartis, Novo Nordisk, Pfizer, and Eli Lilly. RM has received honoraria as a speaker and a board member from Novo Nordisk, Sanofi, Eli Lilly, AstraZeneca, Merck, Novartis and Hikma. FB reports speaker honoraria and travel sponsorship from Bilim Ilaç, Abbott, Novo Nordisk, Sanofi Genzyme, Pfizer, and Eczacibasi Ilaç, and advisory board and consultancy fees from Novo Nordisk, Sanofi Genzyme, Abbott, Pfizer, and Bilim Ilaç. AE has received advisory board fees from AstraZeneca, Boehringer Ingelheim, Merck, and Novo Nordisk, and speaker fees from AstraZeneca, Boehringer Ingelheim, Eli Lilly, Merck, Novartis, Novo Nordisk, and Sanofi. AK has received speaker and advisory board fees from Novo Nordisk, AstraZeneca, Merck & Co., Eli Lilly, Aspen Pharma, Sanofi, Boehringer Ingelheim, Cipla, Mylan, Pharmadynamics, Adcock Ingram, and Pfizer. AH and VR are employees of AstraZeneca.

The remaining author declares that the research was conducted in the absence of any commercial or financial relationships that could be construed as a potential conflict of interest.

This study received funding from AstraZeneca. AstraZeneca as funder had the following involvement with the study: study design, study management as per ICH GCP Sponsor responsibilities guidelines, decision to publish and the preparation of the manuscript. The data collection was performed by local CROs contracted by AstraZeneca and Data analysis was carried out by an independent academic centre (Mid America Heart Institute).

## Publisher’s note

All claims expressed in this article are solely those of the authors and do not necessarily represent those of their affiliated organizations, or those of the publisher, the editors and the reviewers. Any product that may be evaluated in this article, or claim that may be made by its manufacturer, is not guaranteed or endorsed by the publisher.
