# Supplementary material for: Prevalence and risk factors of vascular complications in type 2 diabetes mellitus: Results from discover Middle East and Africa cohort
Source: Front Endocrinol (Lausanne). 2022 Aug 9;13:940309. doi: 10.3389/fendo.2022.940309 (PMC9396276; doi:10.3389/fendo.2022.940309)
Supplement: Supplementary file 1 [file Table_1.docx]

## Supplementary Tables

**Table S1: Inclusion and Exclusion Criteria**

| **Inclusion criteria** |
| --- |
| - Diagnosis of type 2 diabetes mellitus - Age ≥18 years - Initiating a second-line therapy (add-on or switching) after first-line oral treatment with monotherapy, dual therapy or triple therapy - Provision of written informed consent |
| **Exclusion criteria** |
| - Type 1 diabetes mellitus - Pregnancy - Initiation of dual therapy after having previously received two different lines of monotherapy (e.g. initiation of a combination of a sulphonylurea and a DPP-4 inhibitor after successive metformin monotherapy and sulphonylurea monotherapy) - Current treatment with chemotherapy or oral or intravenous steroids - Undergoing dialysis or received a renal transplant - First-line treatment with insulin or another injectable agent as they may have severe disease profile. Patients who received short-term initial treatment with insulin followed by oral therapy were eligible if the treatment with insulin lasted no more than 2 weeks and occurred at least 6 months before initiation of second-line therapy - First-line treatment with herbal remedies or natural medicines alone - Participation in an interventional trial - Condition or circumstance that, in the opinion of the investigator, could significantly compromise the 3-year follow-up (e.g. life-threatening co-morbidities, tourist, non-native language speaker or lack of understanding of the local language in countries where interpreter services are not reliably available, psychiatric disturbances, dementia, alcohol or drug abuse) - Unwilling to sign the informed consent form |

DDP-4: dipeptidyl peptidase-4

| **Event, n (%)** | **Algeria**  **(N=291)** | **Bahrain**  **(N= 70)** | **Egypt**  **(N=583)** | **Jordan**  **(N=271)** | **Kuwait**  **(N=51)** | **Lebanon**  **(N=348)** | **Oman**  **(N=31)** | **Saudi Arabia**  **(N=519)** | **South Africa**  **(N=519)** | **Turkey**  **(N=534)** | **Tunisia**  **(N=213)** | **United Arab Emirates**  **(N=95)** |
| --- | --- | --- | --- | --- | --- | --- | --- | --- | --- | --- | --- | --- |
| **Any microvascular disease** | 69 (23.7) | 16 (22.9) | 124 (21.3) | 28 (10.3) | 16 (31.4) | 49 (14.1) | 10 (32.3) | 105 (20.2) | 48 (9.2) | 108 (20.2) | 40 (18.8) | 10  (10.5) |
| CKD | 11 (3.8) | 4 (5.7) | 1 (0.2) | 6 (2.2) | 3 (5.9) | 5 (1.4) | 1 (3.2) | 3 (0.6) | 8 (1.5) | 11 (2.1) | 4 (1.9) | 4 (4.2) |
| Albuminuria | 15 (5.2) | 0 (0.0) | 8 (1.4) | 7 (2.6) | 2 (3.9) | 21 (6.0) | 10 (32.3) | 16 (3.1) | 6 (1.2) | 8 (1.5) | 23 (10.8) | 4 (4.2) |
| Retinopathy | 13 (4.5) | 4 (5.7) | 14 (2.4) | 6 (2.2) | 11 (21.6) | 4 (1.1) | 0 (0.0) | 20 (3.9) | 9 (1.7) | 33 (6.2) | 5 (2.3) | 4 (4.2) |
| Retinal laser photocoagulation | 3 (1.0) | 0 (0.0) | 2 (0.3) | 0 (0.0) | 2 (3.9) | 0 (0.0) | 0 (0.0) | 2 (0.4) | 1 (0.2) | 4 (0.7) | 3 (1.4) | 0 (0.0) |
| Peripheral Neuropathy | 29 (10.0) | 5 (7.1) | 86 (14.8) | 9 (3.3) | 3 (5.9) | 7 (2.0) | 0 (0.0) | 59 (11.4) | 18 (3.5) | 60 (11.2) | 9 (4.2) | 3 (3.2) |
| Autonomic Neuropathy | 1(0.3) | 0 (0.0) | 14 (2.4) | 2 (0.7) | 1 (2.0) | 2 (0.6) | 0 (0.0) | 4 (0.8) | 0 (0.0) | 6 (1.1) | 1 (0.5) | 1 (1.1) |
| ED | 23 (7.9) | 7 (10.0) | 29 (5.0) | 8 (3.0) | 6 (11.8) | 13 (3.7) | 0 (0.0) | 31 (6.0) | 15 (2.9) | 21 (3.9) | 6 (2.8) | 2 (2.1) |
| **Any macrovascular disease** | 18 (6.2) | 9 (12.9) | 55 (9.4) | 33 (12.2) | 6 (11.8) | 40 (11.5) | 1 (3.2) | 51 (9.8) | 55 (10.6) | 86 (16.1) | 18 (8.5) | 5 (5.3) |
| HF | 1 (0.3) | 2 (2.9) | 4 (0.7) | 3 (1.1) | 1 (2.0) | 4 (1.1) | 0 (0.0) | 8 (1.5) | 6 (1.2) | 11 (2.1) | 0 (0.0) | 1 (1.1) |
| CAD | 13 (4.5) | 5 (7.1) | 44 (7.5) | 26 (9.6) | 6 (11.8) | 36 (10.3) | 1 (3.2) | 38 (7.3) | 45 (8.7) | 69 (12.9) | 13 (6.1) | 3 (3.2) |
| Angina | 2 (0.7) | 1 (1.4) | 16 (2.7) | 2 (0.7) | 4 (7.8) | 0 (0.0) | 0 (0.0) | 12 (2.3) | 16 (3.1) | 22 (4.1) | 5 (2.3) | - |
| MI | 9 (3.1) | 0 (0.0) | 7 (1.2) | 7 (2.6) | 2 (3.9) | 6 (1.7) | 0 (0.0) | 8 (1.5) | 30 (5.8) | 17 (3.2) | 1 (0.5) | - |
| PCI | 3 (1.0) | 3 (4.3) | 14 (2.4) | 12 (4.4) | 4 (7.8) | 12 (3.4) | 1 (3.2) | 17 (3.3) | 7 (1.3) | 33 (6.2) | 6 (2.8) | 1 (1.1) |
| CABG | 2 (0.7) | 1 (1.4) | 3 (0.5) | 0 (0.0) | 0 (0.0) | 7 (2.0) | 0 (0.0) | 3 (0.6) | 8 (1.5) | 11 (2.1) | 0 (0.0) | 1 (1.1) |
| Stroke | 2 (0.7) | 2 (2.9) | 3 (0.5) | 5 (1.8) | 0 (0.0) | 2 (0.6) | 0 (0.0) | 12 (2.3) | 2 (0.4) | 6 (1.1) | 3 (1.4) | 2 (2.1) |
| TIA | 3 (1.0) | 2 (2.9) | 6 (1.0) | 2 (0.7) | 0 (0.0) | 0 (0.0) | 0 (0.0) | 4 (0.8) | 0 (0.0) | 2 (0.4) | 2 (0.9) | 1 (1.1) |
| Carotid stenting | 0.0 (0.0) | 0.0 (0.0) | 1 (0.2) | 1 (0.4) | 0 (0.0) | 0 (0.0) | 0 (0.0) | 2 (0.4) | 0 (0.0) | 0 (0.0) | 0 (0.0) | - |
| Carotid Endarterectomy | 0.0 (0.0) | 0.0 (0.0) | 0 (0.0) | 0 (0.0) | 0 (0.0) | 0 (0.0) | 0 (0.0) | 0 (0.0) | 0 (0.0) | 0 (0.0) | 1 (0.5) | - |
| PAD | 1 (0.3) | 0.0 (0.0) | 7 (1.2) | 2 (0.7) | 0 (0.0) | 1 (0.3) | 0 (0.0) | 0 (0.0) | 3 (0.6) | 4 (0.7) | 0 (0.0) | - |
| Diabetic foot | 2 (0.7) | 2 (2.9) | 2 (0.3) | 2 (0.7) | 0 (0.0) | 1 (0.3) | 0 (0.0) | 0 (0.0) | 2 (0.4) | 10 (1.9) | 0 (0.0) | - |
| Amputation | 1 (0.3) | 0 (0.0) | 1 (0.2) | 0 (0.0) | 0 (0.0) | 2 (0.6) | 0 (0.0) | 0 (0.0) | 5 (1.0) | 3 (0.6) | 0 (0.0) | - |
| Defibrillator use | 0 (0.0) | 0 (0.0) | 0 (0.0) | 0 (0.0) | 0 (0.0) | 0 (0.0) | 0 (0.0) | 0 (0.0) | 0 (0.0) | 0 (0.0) | 0 (0.0) | - |

**Table S2: Crude Prevalence of Microvascular and Macrovascular Complications and Related Procedures at Baseline, Country-wise**

CAD: coronary artery disease; CABG: coronary artery bypass graft; CKD: chronic kidney disease; ED: erectile dysfunction; HF: heart failure; MI: myocardial infarction; PAD: peripheral artery disease; PCI: percutaneous coronary intervention; TIA: transient ischaemic attack
